# Interglomerular Connectivity within the Canonical and GC-D/Necklace Olfactory Subsystems

**DOI:** 10.1371/journal.pone.0165343

**Published:** 2016-11-30

**Authors:** Cedric R. Uytingco, Adam C. Puche, Steven D. Munger

**Affiliations:** 1 Department of Anatomy and Neurobiology, University of Maryland School of Medicine, Baltimore, Maryland, United States of America; 2 Program in Neuroscience, University of Maryland School of Medicine, Baltimore, Maryland, United States of America; 3 Center for Smell and Taste, University of Florida, Gainesville, Florida, United States of America; 4 Department of Pharmacology and Therapeutics, University of Florida, Gainesville, Florida, United States of America; 5 Department of Medicine, Division of Endocrinology, Diabetes and Metabolism, University of Florida, Gainesville, Florida, United States of America; University of Queensland, AUSTRALIA

## Abstract

The mammalian main olfactory system contains several subsystems that differ not only in the receptors they express and the glomerular targets they innervate within the main olfactory bulb (MOB), but also in the strategies they use to process odor information. The canonical main olfactory system employs a combinatorial coding strategy that represents odorant identity as a pattern of glomerular activity. By contrast, the "GC-D/necklace" olfactory subsystem—formed by olfactory sensory neurons expressing the receptor guanylyl cyclase GC-D and their target necklace glomeruli (NGs) encircling the caudal MOB—is critical for the detection of a small number of semiochemicals that promote the acquisition of food preferences. The formation of these socially-transmitted food preferences requires the animal to integrate information about two types of olfactory stimuli: these specialized social chemosignals and the food odors themselves. However, the neural mechanisms with which the GC-D/necklace subsystem processes this information are unclear. We used stimulus-induced increases in intrinsic fluorescence signals to map functional circuitry associated with NGs and canonical glomeruli (CGs) in the MOB. As expected, CG-associated activity spread laterally through both the glomerular and external plexiform layers associated with activated glomeruli. Activation of CGs or NGs resulted in activity spread between the two types of glomeruli; there was no evidence of preferential connectivity between individual necklace glomeruli. These results support previous anatomical findings that suggest the canonical and GC-D/necklace subsystems are functionally connected and may integrate general odor and semiochemical information in the MOB.

## Introduction

The mammalian olfactory system is comprised of multiple subsystems [1]. Canonical olfactory sensory neurons (OSNs) in the mouse main olfactory system (MOS), which respond to a large variety of volatile chemostimuli, express one of approximately a thousand seven-transmembrane odorant receptors along with components of a cAMP-mediated sensory transduction cascade [1]. The axons of canonical OSNs terminate within glomeruli across most of the main olfactory bulb (MOB), where they make synaptic contact with local interneurons and projection neurons [2, 3]. However, the MOS also contains several non-canonical OSNs that can be distinguished from canonical OSNs by the chemostimuli to which they respond, the receptors and signaling proteins they use to transduce those stimuli and the connections they make to the MOB [1]. For example, OSNs expressing the trace amine-associated receptors (TAARs) appear to otherwise engage the canonical cAMP signaling pathway [4]. The axons of TAAR-expressing OSNs target a few glomeruli in a delimited region of the dorsal MOB [5]. By contrast, OSNs expressing the type D receptor guanylyl cyclase (GC-D) utilize a cGMP-mediated signaling mechanism to transduce a small number of social chemostimuli [6–8]. GC-D-expressing (GC-D+) OSNs also display a unique projection pattern into the central nervous system, terminating in over a dozen “necklace” glomeruli that encircle the caudal MOB [1, 9].

**Fig 1:**
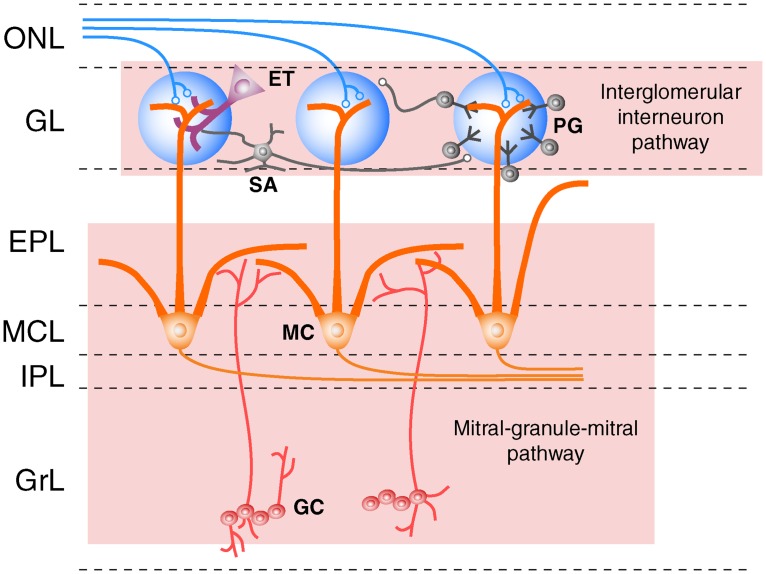
Interglomerular pathways of the main olfactory bulb circuit. Schematic of the main olfactory bulb circuit with the (top) interglomerular-interneuron and (bottom) mitral-granule-mitral pathways highlighted. ONL, olfactory nerve layer; GL, glomerular layer; EPL, external plexiform layer; MCL, mitral cell layer; IPL, internal plexiform layer; GrL, granule cell layer; PG, periglomerular cell; SA, short axon cell; ET, external tufted cell; MC, mitral cell; GC, granule cell.

In the canonical MOS, odorant information is processed by lateral interglomerular-interneuron and mitral-granule-mitral pathways [10, 11] (Fig 1). The interglomerular-interneuron pathway is composed of the projections of short axon neurons in the glomerular layer (GL) [10, 12], while the mitral-granule-mitral pathway involves dendrodendritic synapses between granule cells and mitral cell lateral dendrites within the external plexiform layer (EPL) [11, 13, 14] (Fig 1). Both pathways participate in the lateral center-surround inhibition of neighboring glomerular circuits [10, 11]. This suppression of neighboring circuits and neurons is a fundamental strategy used by other sensory systems as a method of enhancing the contrast of patterned input [15–18]. While we have a growing understanding of the functional connectivity between canonical glomeruli (CGs), it is unknown whether non-canonical glomeruli in the MOB such as the GC-D+ OSN-innervated necklace glomeruli (NGs) utilize similar strategies for interglomerular processing of olfactory information.

The GC-D/necklace subsystem mediates a powerful type of food-related social learning, such as seen during the behavior known as the social transmission of food preference [7]. This behavior depends on the simultaneous detection of a food odor by the canonical MOS and one of a few specific semiochemicals, including carbon disulfide and the peptides guanylin and uroguanylin, by GC-D+ OSNs. Mice lacking an intact chemosensory transduction cascade in GC-D+ OSNs fail to acquire the preference [7, 19]. Neuronal tracing from individual NGs suggest extensive interglomerular connections with other NGs, as well as with nearby and distant CGs [7, 19], suggesting that NGs could be an integration site for semiochemical and general odor information. However, it remains unknown whether the anatomical connections of NGs reflect a similar functional connection with other NGs and CGs. Here, we utilized stimulus-induced increases in intrinsic fluorescent signals, along with microsurgical and pharmacological disruptions of neural pathways, to map functional circuits in the olfactory bulb associated with single, identified canonical and necklace glomeruli and assess the potential for GC-D+ OSN-mediated semiochemical signals to be integrated and/or processed with general odor information as early as the first glomerular synapses.

## Materials and Methods

### Animals

Olfactory bulbs were harvested from 3–6 week old male and female C57BL6/J mice or B6;129P2-*Gucy2d*^*tm2Mom*^/MomJ (GCD-EGFP) mice, the latter which express a tau-EGFP fusion protein after an IRES element placed downstream of the native stop codon for *Gucy2d* (the gene encoding GC-D). All studies were approved by the University of Maryland, Baltimore IACUC committee.

### Olfactory bulb slice preparation

Olfactory bulb slices were prepared as described in the accompanying paper Uytingco *et al*. (accompanying manuscript, DOI: 0165342). Briefly, animals were anesthetized with saturated isoflurane vapor, decapitated, and the olfactory bulbs surgically removed and placed in 4°C oxygenated sucrose-artificial cerebrospinal fluid (sucrose-ACSF) containing 26 mM NaHCO_3_, 1 mM NaH_2_PO_4_, 3 mM KCl, 5 mM MgSO_4_, 0.5 mM CaCl_2_, 10 mM glucose, and 248 mM sucrose, equilibrated with 95% O_2_-5% CO_2_, pH 7.38. Horizontal slices (380–400 μm thick) were cut with a Leica VT1000 vibratome. Slices were incubated in oxygenated ACSF (containing 124 mM NaCl, 26 mM NaHCO_3_, 3 mM KCl, 1.25 mM NaH_2_PO_4_, 2 mM MgSO_4_, 2 mM CaCl_2_, and 15 mM glucose equilibrated with 95% O_2_-5% CO_2_, pH 7.4) at 30°C for 20–30 min then at room temperature (22°C) in ACSF for ~1 hr prior to use. For imaging sessions, individual slices were transferred to a custom recording chamber and perfused with ACSF maintained at a constant 30°C (Bipolar Temperature Controller, Norfolk, VA) at a rate of 2.5 ml/min.

For surgical microcut experiments, horizontal MOB slices were held in a petri dish lined with 1% agar. Surgical mircocuts were delivered through the olfactory nerve layer (ONL), glomerular layer (GL), and/or external plexiform layer (EPL) using a 15°-angled stab knife (Premier Edge) under a dissecting microscope a strategy first implemented by Aungst *et al*. [10]. All surgical microcut procedures were performed on slices submerged in oxygenated ACSF (prior to the 30°C incubation step indicated above). Angled coronal MOB slices were prepared in order to incorporate the necklace glomeruli within the caudal MOB. Isolated brains were vertically cut 60° from the MOB ventral surface, with the cut surface placed on the vibratome stage and cut from the ventral to dorsal surface of the MOB. Resulting coronal slices were incubated in oxygenated ACSF under the same conditions as the horizontal MOB slices. In some experiments, drugs or other compounds were added to the ACSF solution and perfused into the chamber. The pharmacological agent, substrate change, or washouts were perfused into the chamber for 15 min prior to the start of each recording. Pharmacological agents used for the experiments include the GABA_A_ receptor antagonist 4-[6-imino-3-(4-methoxyphenyl)pyridazin-1-yl] butanoic acid hydrobromide (gabazine, GBZ).

### Stimulation protocol

Single glomeruli were stimulated as described in the accompanying paper Uytingco *et al*. (accompanying manuscript, DOI: 0165342). Briefly, single target glomeruli were electrically stimulated at the apical border, within 10–20 μm of the olfactory nerve layer, using a 5 μm tip, pulled theta glass electrode. Unless otherwise indicated, glomeruli received a train stimulus of 100 μA, 50 Hz (pulses/s), 1 ms pulse width, with a train duration of 2 s. This baseline stimulation parameter yielded the most robust and reproducible intrinsic response. All stimulation protocols were delivered through a Cygnus stimulus-isolating unit and driven by a Cygnus PG4000 digital stimulator. For all microcut experiments, test recordings from rostral and caudal stimulations were performed to ensure glomerular circuit integrity. For microcut recordings, the stimulus electrode was placed on the caudal side of the microcut to reduce the potential of in passage nerve fiber stimulation on the lateral signal spread. NGs were identified based on the presence of GFP. Unless otherwise indicated, MOB slices received a standard train stimulus (1 s) of 100 μA, 50 Hz (pulses/s), 1 ms pulse width.

### Intrinsic flavoprotein and NAD(P)H imaging

Imaging of flavoprotein and NAD(P)H fluorescence was performed as described in the accompanying paper Uytingco *et al*. (accompanying manuscript, DOI: 0165342). Briefly, images were collected using a Retiga EXi CCD camera (QImaging) attached to an epifluorescent microscope (Olympus BX51WI) equipped with a 10x water immersion objective lens (0.30 NA) or a 4x lens (0.13 NA) with a customized water immersion adaptor. Standard emission/excitation cubes optimal for flavoprotein (excitation: 430–500 nm; emission: 520–590 nm) or NAD(P)H (excitation: 300–400 nm; emission: 430–500 nm) were used. In order to limit potential phototoxic effects of 100W mercury lamp light exposure, neutral density filters were installed in the illumination path. For flavoprotein imaging, no filter was used for imaging at 4x, while a 0.3 optical density filter was used for imaging at 10x. To limit the more harmful 300-400nm excitation light in NAD(P)H imaging, 0.3 and 0.6 optical density filters were used for imaging at 4x and 10x, respectively. All images were acquired through the Matlab Image Acquisition Toolbox (Mathworks, Matlab v2011b) at 2 frames/s (500 ms exposure) at 360x260 pixel (px) resolutions (using 4x4 hardware binning). The images were subsequently processed and analyzed on a pixel-by-pixel basis using the Matlab Image Processing Toolbox (Mathworks, Matlab v2011b) using script modified from Theyel and colleagues [20]. The first 10 pre-stimulus frames were used to determine the relative fluorescence intensity (ΔF/F) or [(F_n_–F_m_)/F_m_] for each recording trial. For each stimulus parameter or treatment condition, 3–5 repeated trials were averaged prior to signal quantification. Nonspecific background fluorescence and fluorescent bleaching were removed by subtracting non-stimulus ΔF/F recordings from the stimulus ΔF/F recordings. For all displayed images, the processed ΔF/F images were overlaid on top of the static raw fluorescent image. The pixel value for the ΔF/F images were scaled and cutoff at minimum 0% and maximum 4–5% ΔF/F for intrinsic flavoprotein imaging. For intrinsic NAD(P)H imaging, ΔF/F images were scaled and cutoff at minimum -4% and maximum 0% ΔF/F, represented in inverse colors.

### Data analysis

Flavoprotein and NAD(P)H signal analyses were performed after repeated sweep recordings were averaged and background subtracted. Point region of interests (ROIs) were 5x5 pixels (50x50μm) for recordings at 4x magnification. Glomerular layer (GL) ROIs were positioned at the center of the stimulated glomerulus. External plexiform layer (EPL) ROIs were directly below the glomerular ROI and midway between the apical and basal EPL borders. For neighboring ROI analyses, ROIs were selected 360 μm (center to center) either rostral or caudal to the central GL or EPL ROIs. Signal amplitudes, full duration at half-maximal (FDHM) was determined based on the GL and EPL ROI traces, using a custom MATLAB script. When available, the analyses were performed on both the stimulus-dependent light and dark phases. Unless otherwise specified, repeated measures analysis of variance (RM-ANOVA) was performed for all experimental conditions followed by Tukey pairwise comparison post-hoc test. All statistical analyses were performed in Sigmaplot (Sigmaplot v12). Vertical and horizontal heat maps were generated using vertical and horizontal ROIs at 5 px (50 μm) width. Horizontal ROI traces were generated by taking the mean signal response across the 2 s stimulation period. Signal full width at half-maximal (FWHM) was generated by averaging the horizontal ROI traces during the 2 s stimulation period.

## Results

### MOB activity is controlled by GABA feedback

**Fig 2:**
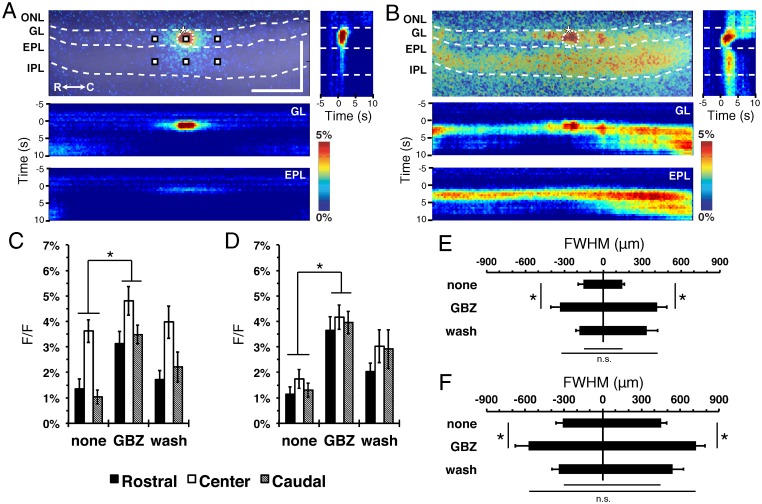
GABA_A_R antagonist enhances lateral flavoprotein signal spread in the GL and EPL. (**A and B**) Representative stimulus-dependent flavoprotein response before treatment (A) and during bath application of 10 μM GBZ (B). (top) Representative overlaid, pseudo-colored ΔF/F images at 2.5s after stimulus initiation (stimulation initiated at 0s). Representative point ROIs (regions of interest; white squares) from stimulated glomerulus, central EPL and neighboring rostral/caudal areas. ONL, olfactory nerve layer; GL, glomerular layer; EPL, external plexiform layer; MCL, mitral cell layer; IPL, internal plexiform layer; GrL, granule cell layer; R, rostral; C, caudal. Asterisk, tip of the stimulating electrode. Targeted glomerulus indicated by dashed circle. Scale bars = 400 μm. (A and B, right) Heat map of the vertical ROI signal response over time. The late flavoprotein signal seen at the top of the image comes from the targeted glomerulus as visualized through the lateral aspect of the MOB slice. (A and B, bottom) Heat maps of the horizontal ROI signal response from the GL and EPL over time. **(C and D)** Mean signal peak of the (C) GL (F_(2,16)_ = 49.2, p = 0.002, n = 9) and (D) EPL (F_(2,16)_ = 5.4, p<0.001, n = 9) at 360 μm rostral (black), center (white), and 360 μm caudal (gray) areas as indicated in (A). **(E and F)** Following GBZ treatment, the mean FWHM lateral spread in the (E) GL (F_(2,8)_ = 14.0, p = 0.018, n = 9) and (F) EPL (F_(2,8)_ = 21.2, p = 0.009, n = 9) increased when compared to pretreated slices. Differences in the duration of the flavoprotein signal in the rostral and caudal dimensions were not quantified.

Transsynaptic stimulus-dependent spread of intrinsic fluorescence signals in the MOB is consistent with the known circuitry associated with CGs (Uytingco *et al*., accompanying manuscript; [21] (Fig 1). Electrical stimulation of single CGs in horizontal MOB slices produce translaminar spread of fluorescence within the MOB as well as lateral spread across both the GL and EPL (Fig 2A). The lateral flow of olfactory information within the MOB depends on both excitatory and inhibitory interneurons within the GL and EPL [12, 22, 23]. To isolate the contribution of excitatory components, MOB slices were treated with 10 μM gabazine (GBZ), a GABA_A_ receptor antagonist. GBZ treatment enhanced the stimulus-dependent signal spread across the MOB layers, and laterally across the GL and EPL (Fig 2B).

ROIs taken from the stimulated glomerulus and the neighboring rostral/caudal glomeruli (Fig 2A) demonstrated enhanced stimulus-dependent response amplitude in the GL areas following GBZ treatment (F_(2,16)_ = 49.2, p = 0.002, n = 9; Fig 2C). A similar increase in response amplitude was observed from the EPL areas directly below the GL ROIs (F_(2,16)_ = 5.4, p<0.001, n = 9; Fig 2D). There was also an increase in stimulus-dependent signal spread. Calculated full-width at half-maximal (FWHM) spread at the GL (F_(2,8)_ = 14.0, p = 0.018, n = 9; Fig 2E) and EPL (F_(2,8)_ = 21.2, p = 0.009, n = 9; Fig 2F) increased two-fold in the presence of GBZ. Despite the increase in signal amplitude, we observed no difference between rostral and caudal lateral signal spread during GBZ treatment. This symmetry is consistent with our earlier observations (Uytingco et al., accompanying manuscript, DOI: 0165342). Taken together, these data show both amplitude and spatial spread of bulbar responses to sensory nerve input are normally under potent GABAergic modulation.

### The interglomerular-interneuron pathway is necessary for GBZ-enhanced lateral spread

There are two known lateral pathways in the MOB: the interglomerular short axon cell network in the GL [10, 12] and the mitral-granule-mitral circuit in the EPL [13, 14]. Past studies used surgical microcuts on bulb slices to isolate the individual pathways to investigate lateral circuit impact on individual recorded neurons [10, 24, 25]. In order to investigate the impact of these circuits on the entire population activity of the glomerular or granule networks, we utilized the same microcut strategy but instead imaged the metabolically-dependent lateral intrinsic signals.

**Fig 3:**
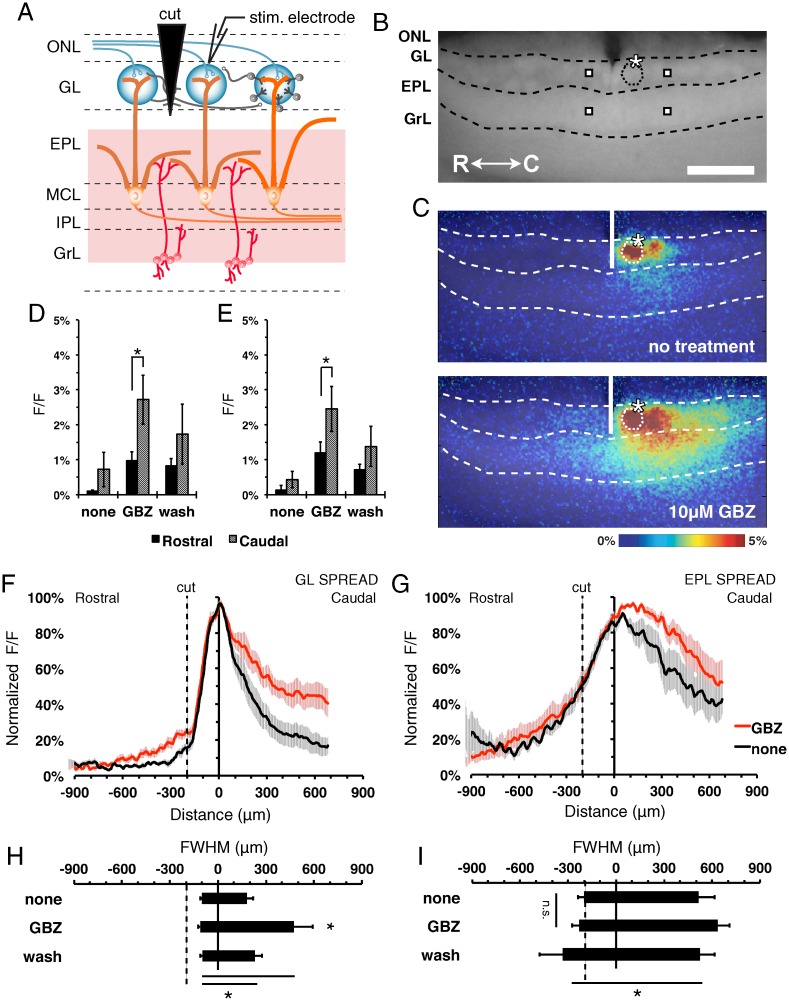
Lateral signal spread is dependent on interglomerular pathway. **(A)** Schematic of the MOB circuitry with GL surgical microcut, and preservation of the mitral-granule-mitral pathway (red highlights). ONL, olfactory nerve layer; GL, glomerular layer; EPL, external plexiform layer; MCL, mitral cell layer; IPL, internal plexiform layer; GrL, granule cell layer; PG, periglomerular cell; SA, short axon cell; ET, external tufted cell; MC, mitral cell; GC, granule cell. **(B)** Fluorescence image of MOB slice. Asterisk, tip of the stimulating electrode. Targeted glomerulus indicated by dashed circle. Representative point ROIs from neighboring rostral/caudal areas (squares). Scale bar = 400 μm. **(C)**. Overlaid pseudo-colored ΔF/F stimulus-dependent responses before (top) and during (bottom) GBZ treatment at 2.5s after stimulus initiation. **(D and E)** Mean signal peak of the GL (D) (F_(2,6)_ = 10.02, p = 0.012, Bonferroni t-test, n = 4) and EPL (E) (F_(2,6)_ = 9.72, p = 0.013, Bonferroni t-test, n = 6) at 360 μm rostral (black) and 360 μm caudal (gray) of the stimulated glomerulus. **(F and G)** Normalized horizontal signal traces in the GL (F) and EPL (G), before (black) and during (red) GBZ treatment. **(H and I)** Mean FWHM signal spread in the GL (H) (F_(2,5)_ = 7.2, p<0.001, n = 6) and EPL (I) (F_(2,5)_ = 0.7, p = 0.02, n = 6).

To examine the contribution of the interglomerular pathway in the lateral spread, a surgical microcut was performed on the GL (Fig 3A and 3B). Electrical stimulation of the glomerulus caudal to the microcut generated a robust and asymmetric signal spread across the GL and EPL. Stimulus-dependent signal spread was observed caudal to the microcut in both the GL and EPL, but rostral to the microcut within the EPL only (Fig 3C). The amplitude and distance of the asymmetrical spread was enhanced during GBZ treatment (Fig 3C). Quantification of the neighboring GL ROIs demonstrated a larger response amplitude on the caudal versus the rostral side of the microcut during GBZ treatment (F_(2,6)_ = 10.02, p = 0.012, Bonferroni t-test, n = 4; Fig 3D). This asymmetrical response was also observed across the EPL (F_(2,6)_ = 9.72, p = 0.013, Bonferroni t-test, n = 6; Fig 3E), suggesting that much of the EPL responses must be due to lateral transmission of activity within the GL. To examine this lateral spread, horizontal ROIs were taken from the GL and EPL. Normalized traces from the GL and EPL (Fig 3F and 3G) indicated failure of stimulus-dependent response spread across the GL microcut under all conditions. GBZ treatment did not alter activity spread within the EPL in regions deep to the other side of the microcut (Fig 3G). Quantification of the FWHM in recordings of both untreated and GBZ-treated tissue shows asymmetrical signal spread in the GL (F_(2,5)_ = 7.2, p<0.001, n = 6; Fig 3H) and EPL (F_(2,5)_ = 0.7, p = 0.02, n = 6; Fig 3I) upon stimulation of glomeruli caudal to the microcut. Together, these results demonstrate that interglomerular circuitry is necessary for the spread of neuronal activity through the GL. They also indicate that a considerable amount of the activity in the EPL is dependent upon activity spread within the GL.

### Lateral activity spread in both the GL and EPL is dependent on reciprocal interactions between the layers

**Fig 4:**
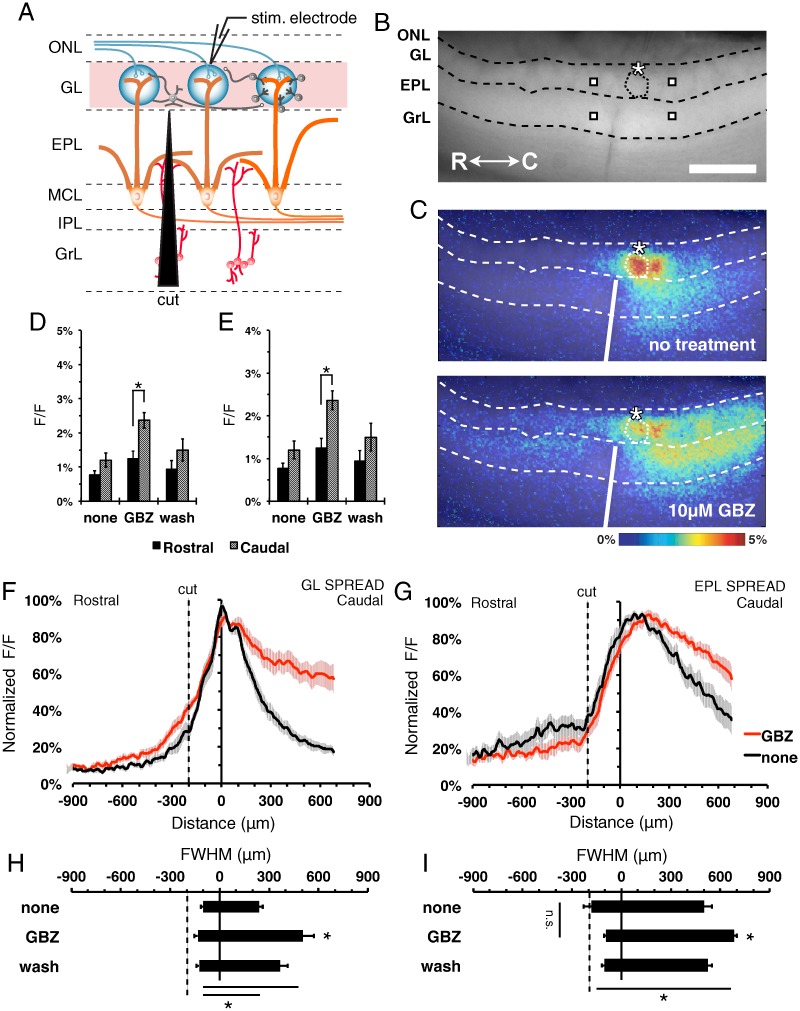
Lateral signal spread is dependent on the mitral-granule-mitral pathway. **(A)** Schematic of the MOB circuitry with EPL surgical microcut, and preservation of the interglomerular-interneuron pathway (red highlights). ONL, olfactory nerve layer; GL, glomerular layer; EPL, external plexiform layer; MCL, mitral cell layer; IPL, internal plexiform layer; GrL, granule cell layer; PG, periglomerular cell; SA, short axon cell; ET, external tufted cell; MC, mitral cell; GC, granule cell. **(B)** Fluorescence image of MOB slice indicating MOB layers, microcut location. Asterisk, tip of the stimulating electrode. Targeted glomerulus indicated by dashed circle. Representative point ROIs from neighboring rostral/caudal areas (squares). Scale bar = 400μm. **(C)** Overlaid pseudo-colored ΔF/F stimulus-dependent responses before (top) and during GBZ treatment (bottom) at 2.5s after stimulus initiation. **(D and E)** Mean signal peak of the (D) GL (F_(2,12)_ = 8.32, p = 0.005, Bonferroni t-test, n = 7) and (E) EPL (F_(2,12)_ = 14.85, p<0.001, Bonferroni t-test, n = 7) at 360 μm rostral (black) and 360 μm caudal (gray) of the activated glomerulus. **(F and G)** Normalized horizontal signal traces in the GL (F) and EPL (G), before (black) and during (red) GBZ treatment. **(H and I)** Mean FWHM signal spread in the (H) GL (F_(2,8)_ = 12.6, p<0.001, n = 7) and (I) EPL (F_(2,8)_ = 6.6, p<0.001, n = 7).

To examine the spread of activity within the mitral-granule-mitral pathway, we examined stimulus-dependent intrinsic signal spread in horizontal MOB slices receiving an isolated EPL surgical microcut (Fig 4A and 4B). Stimulus-dependent signal spread was observed across the microcut in the GL, but not the EPL (Fig 4C). This asymmetrical spread was enhanced following GBZ treatment (Fig 4C). ROI amplitudes from neighboring GL areas (F_(2,12)_ = 8.32, p = 0.005, Bonferroni t-test, n = 7; Fig 4D) and EPL (F_(2,12)_ = 14.85, p<0.001, Bonferroni t-test, n = 7; Fig 4E) demonstrated a larger response amplitude on the caudal versus rostral side of the microcut during GBZ treatment. GBZ treatment did not enhance signal spread across the microcut in the GL or EPL (Fig 4F and 4G). Similar to the GL microcut, quantification of the FWHM in recordings of both untreated and GBZ-treated tissue supported an asymmetrical signal spread in the GL (F_(2,8)_ = 12.6, p<0.001, n = 7; Fig 4H) and EPL (F_(2,8)_ = 6.6, p<0.001, n = 7; Fig 4I). Thus, the EPL microcut prevented spread across the EPL and impacted the spread within the GL. The lateral spread of activity within the GL depends on intact lateral communication within the EPL and vice versa, as shown above. This suggests a reciprocal interaction between circuit elements in both layers is required for normal lateral signal spread in the bulb.

### Necklace glomeruli are functionally interconnected with canonical glomeruli

**Fig 5:**
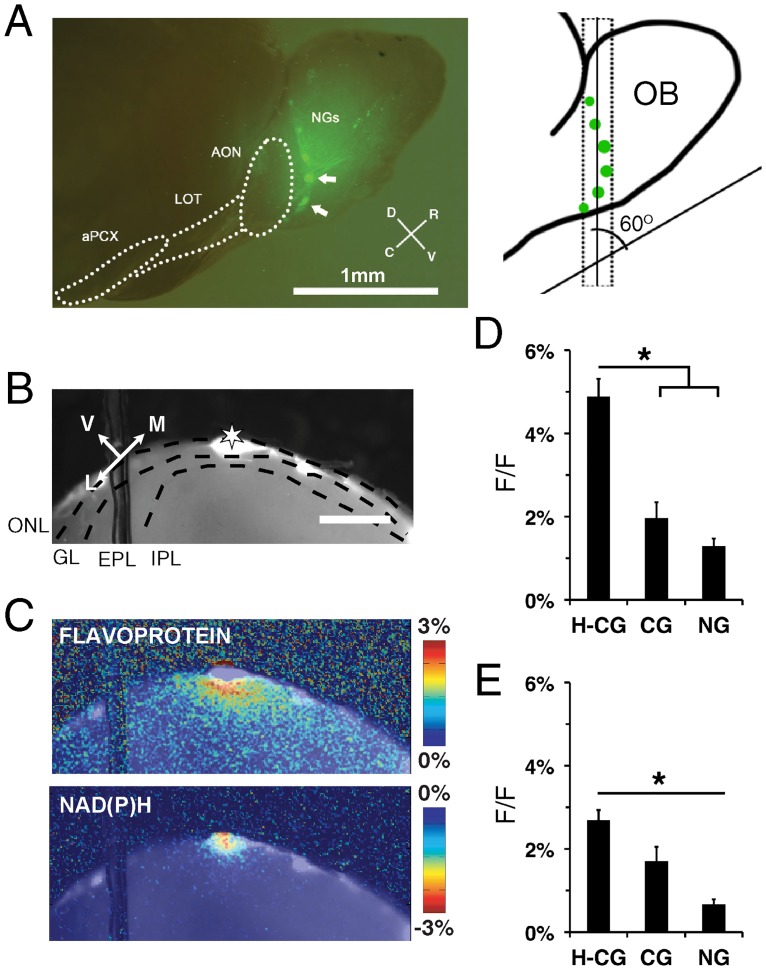
Mapping of intrinsic flavoprotein and NAD(P)H fluorescence signals following necklace glomerulus stimulation. **(A)** (left) Necklace glomeruli (NGs) innervated by GC-D+ OSNs (arrows) from *Gucy2d-GFP* mice. Anterior olfactory nucleus, AON; lateral olfactory tract, LOT; anterior piriform cortex, aPCX. Scale bar = 1 mm. (right) Schematic of angled coronal slice, where the slices were made 60° from the ventral surface of the main olfactory bulb (MOB). **(B)** Fluorescence image of coronal MOB slice, with the stimulus electrode on a single NG (asterisk). Scale bar = 400 μm. **(C)** Representative stimulus-dependent flavoprotein (top) and NAD(P)H (bottom) response at 1.5s after stimulus initiation. **(D and E)** Mean signal peak of the (D) GL (F_(2,60)_ = 5.92, p = 0.004) and (E) EPL (F_(2,66)_ = 5.92, p = 0.002) from stimulated NG, and CGs from coronal slices (C-CG) and horizontal slices (H-CG).

**Fig 6:**
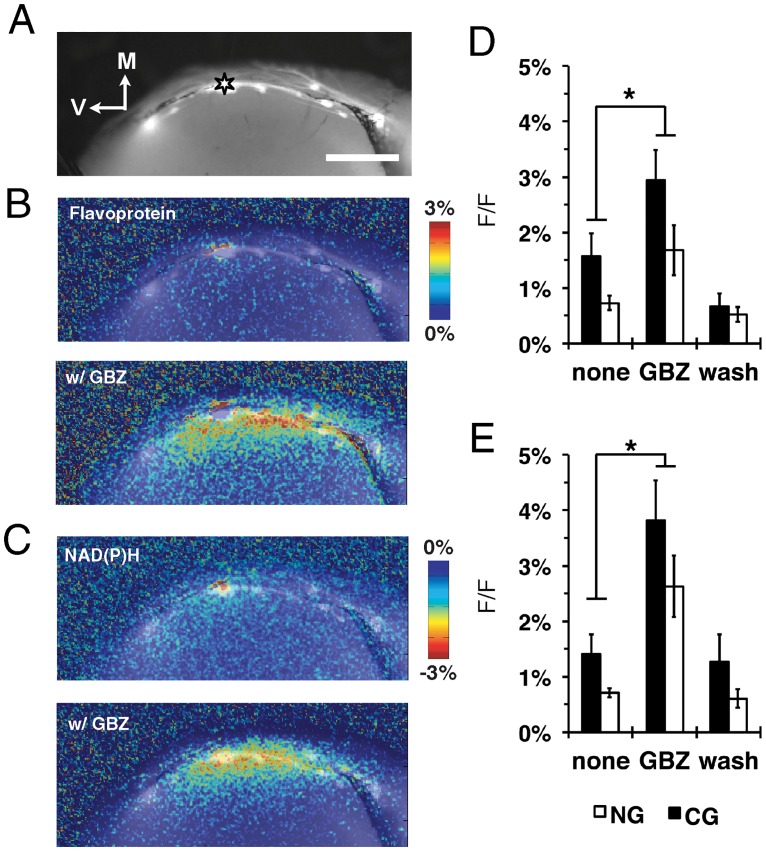
Gabazine treatment enhances lateral signal spread following necklace glomerulus stimulation. **(A)** Fluorescence image of coronal MOB slice with stimulus electrode on a single NG (asterisk). Scale bar = 400μm. **(B and C)** Gabazine (GBZ)-treated slices exhibit increased stimulus-dependent flavoprotein (B) and NAD(P)H (C) signal response and spread. **(D and E)** Mean intrinsic signal peak of the (D) GL (F_(1,2)_ = 18.8, p<0.004) and (E) EPL (F_(1,2)_ = 21.4, p<0.001) from the stimulated necklace (NG) and canonical glomeruli (CG).

Initial characterizations of the NGs hypothesized that these glomeruli form a unique subdomain of the MOB with preferential interglomerular connections [26]. However, tracing studies from individual NGs indicated extensive interglomerular connections to both CGs and other NGs [27]. Even so, the degree to which these anatomical connections reflect functional connections between glomeruli remained unclear. We made angled coronal slices that preserve multiple, adjacent NGs and CGs from the MOBs of mice expressing enhanced green fluorescent protein (GFP) under the control of the *Gucy2d* promoter (GCD-GFP mice) [28] (Fig 5A and 5B). From these slices we recorded stimulus-dependent intrinsic flavoprotein and NAD(P)H (Fig 5C) responses within the NGs and surrounding canonical glomerular areas. Intrinsic signal characteristics following stimulation of either NGs or CGs from the coronal slices had lower GL (F_(2,60)_ = 5.92, p = 0.004; Fig 5D) and EPL (F_(2,66)_ = 5.92, p = 0.002; Fig 5E) amplitudes than those observed in CGs stimulation from more rostral regions of the MOB. The caudal-most region of the MOB has a lower density of cells when observed with Nissl staining, and the reduced intrinsic imaging response was likely due to fewer available cells to serve as sources of intrinsic signals. When we compared the intrinsic signal spread after NG stimulation with or without GBZ, similar to rostral bulbar regions, there was an increase in signal amplitude and spread (Fig 6B and 6C). ROIs of the stimulated CG and NG (F_(1,2)_ = 18.8, p<0.004; Fig 6D) and adjacent EPL (F_(1,2)_ = 21.4, p<0.001; Fig 6E) exhibited increased intrinsic signal amplitude following GBZ treatment. There was also a corresponding increase in lateral spread in the presence of GBZ, indicating activity in these caudal glomeruli are also heavily modulated by GABAergic circuits.

**Fig 7:**
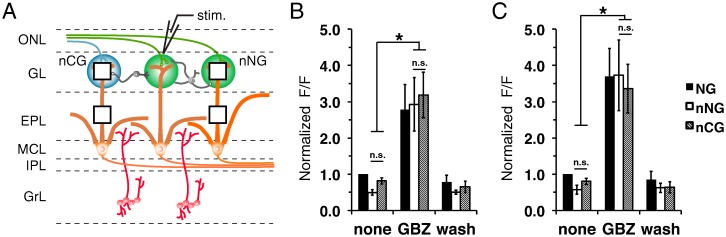
Necklace glomeruli are not preferentially connected to other necklace glomeruli. **(A)** Schematic indicating stimulation of a central necklace glomerulus (NG) and ROIs (squares) taken from the neighboring canonical glomerulus (nCG, blue) and neighboring necklace glomerulus (nNG, green), and their respective EPL areas. ONL, olfactory nerve layer; GL, glomerular layer; EPL, external plexiform layer; MCL, mitral cell layer; IPL, internal plexiform layer; GrL, granule cell layer; PG, periglomerular cell; SA, short axon cell; ET, external tufted cell; MC, mitral cell; GC, granule cell. **(B)** Normalized signal peak from nNG (white) and nCG (gray), following individual necklace glomeruli stimulation. **(C)** Normalized mean signal peak from the EPL below the nNG and nCG.

Next, we quantified the intrinsic signal response of neighboring NGs (nNG) and neighboring CGs (nCG) following individual NG stimulation (Fig 7A) before and after GBZ addition. As shown previously, normalized intrinsic signal amplitude increased in the presence of GBZ in the GL (F_(1,2)_ = 18.8, p<0.001; Fig 7B) and EPL (F_(1,2)_ = 21.4, p<0.001; Fig 7C). However, when we compared normalized amplitudes between the GL (Fig 7B) and EPL areas (Fig 7C) of equidistant nNGs and nCGs following NG stimulation under untreated or GBZ treatment conditions there were no differences. These experiments could not resolve where the signal spread between glomeruli via the GL, EPL or both. The finding that signals from NGs were equally impacting other NGs as well as CGs suggests that there is not preferential functional interconnectivity within the NG subsystem. Rather, the canonical and GC-D/necklace systems interact at the first sites of synaptic integration.

## Discussion

Lateral connectivity within the MOB plays a significant role in olfactory information processing. In this study, we used intrinsic fluorescence imaging and surgical microcuts to examine the functional connectivity between neighboring glomerular circuits that are part of distinct olfactory subsystems. These subsystems have different molecular and anatomic pathways; however, using intrinsic imaging we show that activation of individual NGs resulted in robust lateral spread of activity to both other NGs and CGs without any apparent preferential connectivity. Furthermore, we found that the lateral spread of activity between glomeruli in both the canonical and necklace regions are heavily modulated by GABAergic circuits. Finally, both glomerular and external plexiform circuits must be intact for full lateral spread of activity. This suggests there may be a translaminar reciprocal circuit reinforcing activity within lateral circuits present within the MOB.

### Lateral excitatory pathways and reciprocal connectivity

The interglomerular-interneuron and the mitral-granule-mitral pathways are thought to be the primary interglomerular connections within the MOB. Many studies have indicated that the resulting output of the pathways is lateral inhibition of neighboring glomerular circuits [10, 11]. However, details regarding lateral communicating circuits are still unclear. Voltage-sensitive dye imaging of deafferented MOB surface slices showed widespread depolarization across the GL following individual glomerular stimulation [10]. However, we showed that lateral excitation through the GL requires an intact EPL, and vice versa. This suggests the existence of reciprocal connections between the interglomerular and mitral-granule-mitral pathways. Due to the ubiquitous potential of generating intrinsic responses across neurons, our recordings cannot distinguish between excitatory and inhibitory neurons. But the gross circuit organization and net output could still be interpreted. Known translaminar pathways include the mitral cells themselves, as well as extrabulbar centrifugal fiber pathways and translaminar deep short axon cells. Centrifugal fibers project from several higher brain centers, including the olfactory cortices [29–32]. Centrifugal pathways are thought to participate in a feedback circuit resulting in the increased odorant-evoked inhibition within the MOB [33] and/or the modulation of odorant processing by the secretion of monoamine neurotransmitters [34–39]. However, as these rely on axonal connections from higher brain centers it is unlikely they would participate in a retrograde translaminar synaptic circuit in normal conditions. Alternatively, deep short axon cells may participate in the reciprocal connection, as several cell types extend processes from the IPL across into the EPL and GL [40–42]. The majority of the deep short axon cells express GABA_A_ receptors and voltage-gated K^+^ channel subunits [42] and can target other GABAergic neurons, potentially resulting in disinhibition. While our data indicates the existence of translaminar, mutually-reinforcing activity, the precise glomerular-to-external plexiform circuitry remains to be determined.

### Mapping of intrabulbar functional circuitry associated with the GC-D/necklace subsystem

The GC-D/necklace subsystem is critical for the acquisition of socially transmitted food preferences in response to the coincident detection of food and social odors. Anatomical studies has suggested that the GC-D/necklace subsystem integrates these two olfactory signals due to the heterogeneous sensory innervation and extensive interglomerular connections of NGs with CGs [27]. However, functional studies surrounding the necklace glomeruli have been limited due to the caudal positioning of the glomeruli and lack of a slice model for physiology studies. In the caudal slice model developed for this study, we showed that gross activity elicited from stimulation of an individual NG resulted in a robust spread of activity to other NGs as well as nearby CGs. We could observe no preferential connectivity between NGs, indicating that bulbar circuitry of the GC-D/necklace subsystem is functionally integrated with the canonical MOB circuitry. Thus, the initial integration of social cues and general odors may occur within the MOB prior to the higher-level processing at the olfactory cortices. This immediate processing of odorant information suggests an innate significance of the GC-D/necklace subsystem, and accelerates the recognition of a safe food source without the risk of trial and error. However, to resolve the specific roles of NGs in processing olfactory information related to food preference acquisition it remains necessary to identify NG-associated interneurons and the cortical targets of NG-associated projection neurons as well as to characterize the contributions of other putative chemosensory receptors expressed in GC-D+ OSNs that could respond to food components [43].

The integration of food and social chemostimuli in the MOB has implications for how semiochemical cues are processed in the context of general odors, such as altering the odorant-evoked output of the neighboring canonical circuits. Anatomically, the distribution of NGs in the caudal MOB allows for the GC-D/necklace subsystem to interact with multiple CG circuits, as well as glomerular clusters that exhibit anatomical and functional topographical organizations [44–49]. Glomeruli on the surface of the MOB exhibit distinct topographical organization, with groups of glomeruli responding to specific odorant molecular functional groups [45–47], along with the broader zonal organization that correspond to the distribution of OSNs on the olfactory epithelium [2, 50]. The ability of the GC-D/necklace subsystem to interact with other glomerular clusters suggests that the initial associations of social and food odors necessary for food preference formation could occur at the level of MOB. Additionally, bulbar circuitry may be involved in saliency in the processing of sensory information and more than a simple contrast enhancing ‘pass through’ circuit relay. While the current study highlights the functional connectivity of the NG circuitry, additional studies are required to to identify the neurons in those circuits and to determine how NGs activity modulates the output of neighboring glomerular neurons.

## References

[1] Munger SD, Leinders-Zufall T, Zufall F. Subsystem organization of the mammalian sense of smell. Annu Rev Physiol. 2009;71:115–40. 10.1146/annurev.physiol.70.113006.100608 18808328

[2] Mombaerts P, Wang F, Dulac C, Chao SK, Nemes A, Mendelsohn M, et al Visualizing an olfactory sensory map. Cell. 1996;87(4):675–86. Epub 1996/11/15. 8929536 10.1016/s0092-8674(00)81387-2

[3] Kasowski HJ, Kim H, Greer CA. Compartmental organization of the olfactory bulb glomerulus. J Comp Neurol. 1999;407(2):261–74. Epub 1999/04/23. 10213094

[4] Liberles SD, Buck LB. A second class of chemosensory receptors in the olfactory epithelium. Nature. 2006;442(7103):645–50. Epub 2006/08/01. 10.1038/nature05066 16878137

[5] Pacifico R, Dewan A, Cawley D, Guo C, Bozza T. An olfactory subsystem that mediates high-sensitivity detection of volatile amines. Cell Rep. 2012;2(1):76–88. Epub 2012/07/31. 10.1016/j.celrep.2012.06.006 22840399 PMC3408605

[6] Leinders-Zufall T, Cockerham RE, Michalakis S, Biel M, Garbers DL, Reed RR, et al Contribution of the receptor guanylyl cyclase GC-D to chemosensory function in the olfactory epithelium. Proceedings of the National Academy of Sciences of the United States of America. 2007;104(36):14507–12. 10.1073/pnas.0704965104 17724338 PMC1964822

[7] Munger SD, Leinders-Zufall T, McDougall LM, Cockerham RE, Schmid A, Wandernoth P, et al An olfactory subsystem that detects carbon disulfide and mediates food-related social learning. Current biology: CB. 2010;20(16):1438–44. 10.1016/j.cub.2010.06.021 20637621 PMC2929674

[8] Zufall F, Munger SD. Receptor guanylyl cyclases in mammalian olfactory function. Mol Cell Biochem. 2010;334(1–2):191–7. Epub 2009/11/27. 10.1007/s11010-009-0325-9 19941039 PMC2809823

[9] Juilfs DM, Fulle HJ, Zhao AZ, Houslay MD, Garbers DL, Beavo JA. A subset of olfactory neurons that selectively express cGMP-stimulated phosphodiesterase (PDE2) and guanylyl cyclase-D define a unique olfactory signal transduction pathway. Proceedings of the National Academy of Sciences of the United States of America. 1997;94(7):3388–95. 9096404 10.1073/pnas.94.7.3388 PMC20380

[10] Aungst JL, Heyward PM, Puche AC, Karnup SV, Hayar A, Szabo G, et al Centre-surround inhibition among olfactory bulb glomeruli. Nature. 2003;426(6967):623–9. Epub 2003/12/12. 10.1038/nature02185 14668854

[11] Schoppa NE, Urban NN. Dendritic processing within olfactory bulb circuits. Trends Neurosci. 2003;26(9):501–6. Epub 2003/09/02. 10.1016/S0166-2236(03)00228-5 12948662

[12] Kiyokage E, Pan YZ, Shao Z, Kobayashi K, Szabo G, Yanagawa Y, et al Molecular identity of periglomerular and short axon cells. J Neurosci. 2010;30(3):1185–96. Epub 2010/01/22. 10.1523/JNEUROSCI.3497-09.2010 20089927 PMC3718026

[13] Buonviso N, Chaput MA, Berthommier F. Similarity of granular-induced inhibitory periods in pairs of neighboring mitral/tufted cells. J Neurophysiol. 1996;76(4):2393–401. Epub 1996/10/01. 8899612 10.1152/jn.1996.76.4.2393

[14] Urban NN, Sakmann B. Reciprocal intraglomerular excitation and intra- and interglomerular lateral inhibition between mouse olfactory bulb mitral cells. The Journal of physiology. 2002;542(Pt 2):355–67. Epub 2002/07/18. 10.1113/jphysiol.2001.013491 12122137 PMC2290433

[15] DeVries SH, Baylor DA. Synaptic circuitry of the retina and olfactory bulb. Cell. 1993;72 Suppl:139–49. Epub 1993/01/01. 8428375 10.1016/s0092-8674(05)80033-9

[16] Kuffler SW. Discharge patterns and functional organization of mammalian retina. J Neurophysiol. 1953;16(1):37–68. Epub 1953/01/01. 13035466 10.1152/jn.1953.16.1.37

[17] Ma X, Suga N. Lateral inhibition for center-surround reorganization of the frequency map of bat auditory cortex. J Neurophysiol. 2004;92(6):3192–9. Epub 2004/11/19. 10.1152/jn.00301.2004 15548634

[18] Sur M. Receptive fields of neurons in areas 3b and 1 of somatosensory cortex in monkeys. Brain Res. 1980;198(2):465–71. Epub 1980/10/06. 6250672 10.1016/0006-8993(80)90762-3

[19] Arakawa H, Kelliher KR, Zufall F, Munger SD. The Receptor Guanylyl Cyclase Type D (GC-D) Ligand Uroguanylin Promotes the Acquisition of Food Preferences in Mice. Chemical senses. 2013;38(5):391–7. 10.1093/chemse/bjt015 23564012 PMC3657734

[20] Theyel BB, Llano DA, Issa NP, Mallik AK, Sherman SM. In vitro imaging using laser photostimulation with flavoprotein autofluorescence. Nat Protoc. 2011;6(4):502–8. Epub 2011/04/02. 10.1038/nprot.2011.315 21455186 PMC4758202

[21] Nagayama S, Homma R, Imamura F. Neuronal organization of olfactory bulb circuits. Front Neural Circuits. 2014;8:98 Epub 2014/09/19. 10.3389/fncir.2014.00098 25232305 PMC4153298

[22] Gire DH, Schoppa NE. Control of on/off glomerular signaling by a local GABAergic microcircuit in the olfactory bulb. J Neurosci. 2009;29(43):13454–64. Epub 2009/10/30. 10.1523/JNEUROSCI.2368-09.2009 19864558 PMC2786286

[23] Hayar A, Ennis M. Endogenous GABA and glutamate finely tune the bursting of olfactory bulb external tufted cells. J Neurophysiol. 2007;98(2):1052–6. Epub 2007/06/15. 10.1152/jn.01214.2006 17567771 PMC2366085

[24] Shirley CH, Coddington EJ, Heyward PM. All-or-none population bursts temporally constrain surround inhibition between mouse olfactory glomeruli. Brain Res Bull. 2010;81(4–5):406–15. Epub 2009/11/17. 10.1016/j.brainresbull.2009.10.022 19913074

[25] Whitesell JD, Sorensen KA, Jarvie BC, Hentges ST, Schoppa NE. Interglomerular lateral inhibition targeted on external tufted cells in the olfactory bulb. J Neurosci. 2013;33(4):1552–63. Epub 2013/01/25. 10.1523/JNEUROSCI.3410-12.2013 23345229 PMC3711647

[26] Shinoda K, Ohtsuki T, Nagano M, Okumura T. A possible functional necklace formed by placental antigen X-P2-immunoreactive and intensely acetylcholinesterase-reactive (PAX/IAE) glomerular complexes in the rat olfactory bulb. Brain research. 1993;618(1):160–6. Epub 1993/07/30. 8402170 10.1016/0006-8993(93)90440-x

[27] Cockerham RE, Puche AC, Munger SD. Heterogeneous sensory innervation and extensive intrabulbar connections of olfactory necklace glomeruli. PloS one. 2009;4(2):e4657 10.1371/journal.pone.0004657 19247478 PMC2645502

[28] Walz A, Feinstein P, Khan M, Mombaerts P. Axonal wiring of guanylate cyclase-D-expressing olfactory neurons is dependent on neuropilin 2 and semaphorin 3F. Development. 2007;134(22):4063–72. 10.1242/dev.008722 17942483

[29] Price JL, Powell TP. An experimental study of the origin and the course of the centrifugal fibres to the olfactory bulb in the rat. J Anat. 1970;107(Pt 2):215–37. Epub 1970/09/01. 5487119 PMC1234020

[30] Price JL, Powell TP. An electron-microscopic study of the termination of the afferent fibres to the olfactory bulb from the cerebral hemisphere. J Cell Sci. 1970;7(1):157–87. Epub 1970/07/01. 5476854 10.1242/jcs.7.1.157

[31] Haberly LB, Price JL. Association and commissural fiber systems of the olfactory cortex of the rat. II. Systems originating in the olfactory peduncle. The Journal of comparative neurology. 1978;181(4):781–807. Epub 1978/10/15. 10.1002/cne.901810407 690285

[32] Haberly LB, Price JL. Association and commissural fiber systems of the olfactory cortex of the rat. The Journal of comparative neurology. 1978;178(4):711–40. Epub 1978/04/15. 10.1002/cne.901780408 632378

[33] Boyd AM, Sturgill JF, Poo C, Isaacson JS. Cortical feedback control of olfactory bulb circuits. Neuron. 2012;76(6):1161–74. Epub 2012/12/25. 10.1016/j.neuron.2012.10.020 23259951 PMC3725136

[34] Shipley MT, Halloran FJ, de la Torre J. Surprisingly rich projection from locus coeruleus to the olfactory bulb in the rat. Brain research. 1985;329(1–2):294–9. Epub 1985/03/11. 3978450 10.1016/0006-8993(85)90537-2

[35] McLean JH, Shipley MT, Nickell WT, Aston-Jones G, Reyher CK. Chemoanatomical organization of the noradrenergic input from locus coeruleus to the olfactory bulb of the adult rat. The Journal of comparative neurology. 1989;285(3):339–49. Epub 1989/07/15. 10.1002/cne.902850305 2547851

[36] McLean JH, Shipley MT. Serotonergic afferents to the rat olfactory bulb: II. Changes in fiber distribution during development. J Neurosci. 1987;7(10):3029–39. Epub 1987/10/01. 3668614 10.1523/JNEUROSCI.07-10-03029.1987 PMC6569192

[37] Won MH, Ohno T, Suh JG, Lee JC, Jo SM, Oh YS, et al Serotonergic neurons are present and innervate blood vessels in the olfactory bulb of the laboratory shrew, Suncus murinus. Neurosci Lett. 1998;243(1–3):53–6. Epub 1998/04/16. 9535111 10.1016/s0304-3940(98)00084-6

[38] Ichikawa T, Hirata Y. Organization of choline acetyltransferase-containing structures in the forebrain of the rat. J Neurosci. 1986;6(1):281–92. Epub 1986/01/01. 3944622 10.1523/JNEUROSCI.06-01-00281.1986 PMC6568606

[39] Ojima H, Yamasaki T, Kojima H, Akashi A. Cholinergic innervation of the main and the accessory olfactory bulbs of the rat as revealed by a monoclonal antibody against choline acetyltransferase. Anat Embryol (Berl). 1988;178(6):481–8. Epub 1988/01/01. 3223607 10.1007/BF00305035

[40] Schneider SP, Macrides F. Laminar distributions of internuerons in the main olfactory bulb of the adult hamster. Brain research bulletin. 1978;3(1):73–82. Epub 1978/01/01. 630423 10.1016/0361-9230(78)90063-1

[41] Eyre MD, Antal M, Nusser Z. Distinct deep short-axon cell subtypes of the main olfactory bulb provide novel intrabulbar and extrabulbar GABAergic connections. J Neurosci. 2008;28(33):8217–29. Epub 2008/08/15. 10.1523/JNEUROSCI.2490-08.2008 18701684 PMC2630517

[42] Eyre MD, Kerti K, Nusser Z. Molecular diversity of deep short-axon cells of the rat main olfactory bulb. The European journal of neuroscience. 2009;29(7):1397–407. Epub 2009/04/07. 10.1111/j.1460-9568.2009.06703.x 19344330

[43] Perez-Gomez A, Bleymehl K, Stein B, Pyrski M, Birnbaumer L, Munger SD, et al Innate Predator Odor Aversion Driven by Parallel Olfactory Subsystems that Converge in the Ventromedial Hypothalamus. Curr Biol. 2015;25(10):1340–6. Epub 2015/05/06. 10.1016/j.cub.2015.03.026 25936549 PMC4439360

[44] Igarashi KM, Ieki N, An M, Yamaguchi Y, Nagayama S, Kobayakawa K, et al Parallel mitral and tufted cell pathways route distinct odor information to different targets in the olfactory cortex. J Neurosci. 2012;32(23):7970–85. Epub 2012/06/08. 10.1523/JNEUROSCI.0154-12.2012 22674272 PMC3636718

[45] Kobayakawa K, Kobayakawa R, Matsumoto H, Oka Y, Imai T, Ikawa M, et al Innate versus learned odour processing in the mouse olfactory bulb. Nature. 2007;450(7169):503–8. Epub 2007/11/09. 10.1038/nature06281 17989651

[46] Mori K, Sakano H. How is the olfactory map formed and interpreted in the mammalian brain? Annu Rev Neurosci. 2011;34:467–99. Epub 2011/04/08. 10.1146/annurev-neuro-112210-112917 21469960

[47] Mori K, Takahashi YK, Igarashi KM, Yamaguchi M. Maps of odorant molecular features in the Mammalian olfactory bulb. Physiol Rev. 2006;86(2):409–33. Epub 2006/04/08. 10.1152/physrev.00021.2005 16601265

[48] Ressler KJ, Sullivan SL, Buck LB. A zonal organization of odorant receptor gene expression in the olfactory epithelium. Cell. 1993;73(3):597–609. Epub 1993/05/07. 7683976 10.1016/0092-8674(93)90145-g

[49] Vassar R, Ngai J, Axel R. Spatial segregation of odorant receptor expression in the mammalian olfactory epithelium. Cell. 1993;74(2):309–18. Epub 1993/07/30. 8343958 10.1016/0092-8674(93)90422-m

[50] Mori K, Nagao H, Yoshihara Y. The olfactory bulb: coding and processing of odor molecule information. Science. 1999;286(5440):711–5. Epub 1999/10/26. 10531048 10.1126/science.286.5440.711

